# Comparative Transcriptome Analysis of Leaves and Roots Responses in Salt-Tolerant Barley Line CC89/Giza123 Under Salinity Stress

**DOI:** 10.3390/cimb48070718

**Published:** 2026-07-14

**Authors:** Muhammad Matloob Javed, Abdullah A. Al-Doss, Muhammad Altaf Khan, Salem S. Alghamdi, Basharat A. Dar, Abdelhalim I. Ghazy

**Affiliations:** Plant Production Department, College of Food & Agriculture Sciences, King Saud University, Riyadh 11451, Saudi Arabia; matloobjaved@gmail.com (M.M.J.); aaldoss@ksu.edu.sa (A.A.A.-D.); salem@ksu.edu.sa (S.S.A.); bdar@ksu.edu.sa (B.A.D.); aghazy@ksu.edu.sa (A.I.G.)

**Keywords:** barley breeding line CC89/Giza123, salinity, transcriptome, differentially expressed gene (DEG), and putative salt-responsive genes

## Abstract

Salinity stress has a debilitating effect on crop productivity and triggers complex molecular changes in plants, and understanding these responses is important for improving stress tolerance. This study investigated transcriptomic changes in the salt-tolerant barley line CC89/Giza123 by analyzing gene expression in roots and leaves following exposure to 200 mM NaCl for 12 and 24 h. The number of differentially expressed genes (DEGs) showed a much stronger response in roots than in leaves. At 12 h, roots showed 1836 DEGs, and this number increased to 2696 at 24 h, whereas leaves showed 256 DEGS at 12 h, and 787 at 24 h. The presence of strong and early activation in roots appears to indicate that roots play a key role in the adaptive response to salinity stress initiation. GO and KEGG analyses of differentially expressed genes revealed tissue- and time-specific responses. Roots showed rapid activation of ribosome and secondary-metabolite pathways at 12 h, followed by shifts toward carbon fixation and energy-related pathways at 24 h. Leaves responded early by adjusting photosynthesis-antenna proteins and later expanded their response to defense-related and amino-acid biosynthesis pathways. Important salt-responsive genes were identified in both tissues, including protein kinases, protein phosphatases 2C (PP2Cs), phospholipases, aquaporins, detoxification enzymes, molecular chaperones, and Late Embryogenesis Abundant (LEA) proteins. These results highlight clear tissue-specific and time-dependent differences in how plants respond to salt stress, providing insights into the metabolic and regulatory mechanisms involved in salt tolerance. Overall, these results provide evidence that the response of barley to salinity is achieved by using coordinated and dynamic molecular changes in different tissues. The transcriptomic dataset is a useful source of candidate genes for further functional studies and is a significant resource for breeding and biotechnological approaches to the production of salt-tolerant cereal crops.

## 1. Introduction

Global agriculture faces mounting threats resulting from population growth, climatic variability, and progressive loss of arable lands, thereby threatening food security [1,2]. By the year 2070, the world population is expected to go beyond the ten billion mark, thereby increasing the need for sustainable crop production systems [3]. Among the most important environmental constraints is soil salinity, which currently impacts more than 800 million hectares globally and is increasing even further with shifts in climate and suboptimal irrigation practices in mostly arid and semi-arid regions [4,5,6].

Salinity stress causes the disturbance of plant growth by inducing osmotic imbalance, ion toxicity and oxidative damage, which ultimately affects photosynthesis and cell division and influences the overall productivity [7,8,9]. Sodium accumulation in the rhizosphere precipitates cellular dehydration and metabolic dysfunction and thereby requires the activation of intricate tolerance mechanisms. Plants react to salinity through a series of physiological, biochemical and molecular adaptations, including the production of osmoprotectants, activation of antioxidant defense systems and ion transporters and signaling cascades [10,11,12,13]. Key molecular players in the processes of salt stress responses include transcription factors like CBF/DREB and MAPKs, ion homeostasis regulators like the SOS pathway, and hormone-mediated signaling networks [14,15].

High-throughput RNA sequencing (RNA-Seq) has become a very valuable tool to break down these responses at the transcriptome level to identify differentially expressed genes (DEGs), regulatory modules, and loci of interest for genetic improvement [16,17]. Salt stress responses have been well studied in several species of plants using RNA sequencing, such as barley [18,19] and Arabidopsis [20]. These investigations have shown the involvement of many salt tolerance genes in a variety of biological processes.

Barley (*Hordeum vulgare* L.) is a valuable global cereal crop, which is an excellent model to study salinity because of its genetic diversity, economic value, and moderate tolerance to salt [21,22]. Recent transcriptomic studies have revealed genotype-specific mechanisms of responding to salt stress, where tolerant cultivars have high levels of expression of osmoprotection, detoxification, and ion transport genes [23,24]. Our previous studies have shown that the barley breeding line CC89/Giza123 shows high tolerance to salinity in the field and controlled conditions [25]. Despite these advances, the complexity of genotype-dependent responses and tissue-specific expressions of these is still insufficiently explored. In order to close this gap in knowledge, the current study examines the transcriptomic response of the salt-tolerant barley breeding line CC89/Giza123 subjected to salt stress using RNA-Seq. By analyzing gene expressions in root and leaf tissue after exposure to 200 mM NaCl for 12 and 24 h, we aim to identify the main differentially expressed genes (DEGs) and functional pathways linked to salinity. The findings will support a better understanding of the molecular basis of salt-stress adaptation and will facilitate the development of salt-stress-tolerant barley cultivars by marker-assisted selection and biotechnology approaches.

## 2. Materials and Methods

### 2.1. Plant Materials and Salt Treatment

To investigate the molecular basis of salt tolerance in barley, we conducted an RNA-Seq experiment using the salt-tolerant barley line CC89/Giza123, which has been previously characterized for its improved physiological responses to salinity stress [25].

Barley seeds line CC89/Giza123 were surface sterilized with 5% sodium hypochlorite solution for 10 min and rinsed with sterile distilled water. The seeds were then germinated in the dark at 25 °C for three days and then placed in an aerated hydroponic system with a standard nutrient solution. Plants were grown in controlled conditions with a photoperiod of 16 h, at a temperature of 22 °C and a relative humidity of ~60%. Salt stress was gradually applied by increasing the NaCl concentration in three increments over three consecutive days: 50 mM on day 1, 50 mM on day 2 (final concentration 100 mM NaCl), and 100 mM on day 3 (final concentration 200 mM NaCl). Root and leaf samples were obtained at three time points: before (0 mM NaCl as control), 12 h after reaching 200 mM NaCl, and 24 h after full stress exposure. The collected roots and leaves were carefully washed, separated, flash frozen in liquid nitrogen, and stored at −80 °C until RNA extraction. Two biological replicates were prepared for each treatment condition and used for transcriptome sequencing to identify differentially expressed genes and pathways associated with salt tolerance.

### 2.2. RNA Extraction and Sequencing

Total RNA was isolated from the root and leaf tissues of control and salt-stressed barley plants using the Total RNA Extraction Kit and following the manufacturer’s protocol (Promega Corporation, Madison, WI, USA). Genomic DNA contamination was removed by treatment with RNase-free DNase I. The integrity and purity of RNA samples were checked by NanoDrop Spectrophotometer (NanoDrop Technologies/Thermo Fisher Scientific, Wilmington, DE, USA) and Agilent Bioanalyzer 21000 (Agilent Technologies, Palo Alto, CA, USA). Only high-quality RNA samples with an RNA Integrity Number (RIN) of more than 7 were used for downstream processing. RNA-Seq libraries were generated by the TruSeq RNA Sample Preparation Kit (Illumina Technologies, San Diego, CA, USA) and sequenced on the Illumina HiSeq 4000 to generate 2 × 150 bp paired-end reads for each sample. Sequencing was performed at the Beijing Genome Institute (Hong Kong) from a total of 12 libraries. Low-quality reads were filtered before the analysis, and biological replicates were grouped accordingly.

### 2.3. Sequencing Data Analysis

Sequencing data were processed using SOAPnuke v1.5.2 (BGI, Shenzhen, China) to remove low-quality reads and contaminants. Specifically, reads that contained adapter sequences, more than 5% ambiguous bases (N), or more than 20% of bases with a quality score of less than 15 were discarded. The resulting high-quality clean reads were saved in FASTQ format for subsequent analysis. For alignment, we used HISAT (Hierarchical Indexing for Spliced Alignment of Transcripts) in order to align the RNA-Seq reads with the reference genome of the barley (Hordeum vulgare, Genome Version: BarleyMorexV2) downloaded from the PGSB barley genome database. HISAT uses a Hierarchical Graph FM index (HGFM) that allows the fast and accurate alignment of spliced transcripts. Following alignment, gene expression levels were determined using sequence alignment tools, and transcriptome-wide analysis was carried out in order to identify differentially expressed genes and pathways involved in the salt stress response in the barley genotype CC89/Giza123.

### 2.4. Transcript Quantification and Differential Gene Expression Analysis

To estimate gene expression levels, clean RNA-Seq reads were aligned to the reference barley transcriptome using Bowtie2 software (version 2.2.5) [26]. Gene and isoform abundances were quantified with the RNA-Seq by Expectation Maximization (RSEM) algorithm [27], which accounts for the uncertainty in the mapping of reads and performs an accurate quantification of expression. Differential expression analysis was conducted with the DESeq2 package in the standard workflow as described by Love et al. [28]. This approach allowed the identification of the differentially expressed genes (DEGs) between control and salt-stressed samples.

### 2.5. Functional Annotation

To functionally characterize the assembled unigenes, we used a series of bioinformatics tools and databases to conduct comprehensive sequence alignment and annotation of unigenes. Initial alignment of all unigene sequences with the non-redundant nucleotide (Nr) database from the National Center for Biotechnology Information (NCBI) was performed using BLASTN (verion 2.15.0). BLASTx (version 2.14.0) was used to align the sequences with the Nr protein, Swiss-Prot, KEGG pathway, and COG databases. These alignments aided in determining sequence orientation and predicting protein-coding regions, with the top-ranked BLASTx hits used as a reference for coding-region inference. Unigenes that did not show any relation with one of the aforementioned databases were further analyzed by ESTScan [29] to determine coding regions and sequence directionality. For the functional annotation, the homologous sequences were identified by using BLASTx searches against the NR database with an E-value threshold of 1 × 10^−5^. The resulting hits were annotated using Blast2GO [30] and InterProScan5 to determine Gene Ontology (GO) terms and protein domain information. GO classifications were further analyzed and visualized using WEGO 2.0 software [31], which has allowed categorisation of unigenes according to biological processes, molecular functions, and cellular components. Additionally, BLASTx analysis against the KEGG pathway database was performed to map unigenes to putative metabolic and signaling pathways that gave information about their functional role in the salt stress response.

### 2.6. GO and KEGG Pathway Enrichment Analysis

Gene Ontology (GO) enrichment analysis was conducted in order to define biologically significant functions for the candidate gene set. This is a statistical approach to compare the frequency of GO terms in the set of target genes with a reference background using, for example, a hypergeometric test. Enrichment results are presented as GO terms with *p*-values and fold-change values, which indicate the statistical link between the gene set and a GO term. GO terms with a false discovery rate (FDR) threshold of Q < 0.05 were considered significantly enriched. To further investigate functional pathways, we performed KEGG pathway enrichment analysis using the Kyoto Encyclopedia of Genes and Genomes [32], an extensive resource for molecular interaction, reaction, and network data. Similar to GO analysis, KEGG enrichment was based on a statistical comparison of gene sets and pathways with Q < 0.05, which were considered to be significantly enriched among differentially expressed genes.

### 2.7. RNA-Seq Data Validation Using Real-Time PCR

Validation of results from RNA-Seq was performed with reverse transcription followed by quantitative real-time PCR (qRT-PCR). Total RNA isolation, quantification, and DNase treatment were performed as previously described. Complementary DNA (cDNA) was synthesized from 1 μg of total RNA, using the GoScript Reverse Transcription System Kit (Promega corporation, Madison, WI, USA), following the manufacturer’s protocol. qRT-PCR was performed using the Power SYBR Green qPCR Master Mix (Applied Biosystems, Life Technologies, Carlsbad, CA, USA) on 96-well plates. Gene-specific primers were designed using Primer3 software (https://primer3.ut.ee/; accessed on 1 July 2026), and the expression levels of 21 randomly selected genes and the internal control gene, Actin, were quantified using an Applied Biosystems Veriti Thermal Cycler. Primer sequences used in the analysis are presented in Supplementary Table S1. Each 20 μL qRT-PCR contained 1 μL 4-fold diluted cDNA, 10 μL 2× SYBR Green PCR buffer, 7 μL nuclease-free water, and 1 μL each primer (10 μM). The thermal cycling conditions were as follows: an initial denaturation of 95 °C for 10 min, 40 cycles of 95 °C for 30 s and 60 °C for 60 s. Amplification data were analyzed by using Step OneTM Software v2.2.2 (Applied Biosystems, Foster City, CA, USA). Relative transcript abundance was computed by the 2^−ΔΔCT^ method, and fold-changes were calculated by comparing expression levels in salt-treated samples to those in untreated controls (0 h).

## 3. Results

### 3.1. RNA Extraction and Data Analysis of RNA-Seq

Next-generation sequencing (NGS) requires RNA of high quality. Traditional extraction procedures are usually time-consuming and need massive amounts of RNA. It also provides an RNA Integrity Number (RIN), which indicates the level of RNA degradation. Each run analyzes up to 12 samples in about 25 min. Prior to the synthesis of cDNA samples, all extracted samples were subjected to Bioanalyzer analysis; only those of high quality were selected for downstream steps. Purity and concentration were also evaluated using a NanoDrop spectrophotometer; only samples with 260/280 and 260/230 ratios near 2.0 and concentrations of more than 100 ng/μL were considered suitable samples. A RIN value of more than 7 was found acceptable for sequencing. For this study, cDNA libraries were prepared from root and leaf tissues of the barley cultivar CC89/Giza123 after exposure to 200 mM NaCl. Samples were harvested at 12 h and 24 h post-treatments, resulting in a total of twelve cDNA libraries. Sequencing was carried out on the Illumina HiSeq 4000 platform. Raw reads were subject to stringent quality control to remove low-quality sequences, adapter contamination, and reads with high percentages of ambiguous nucleotides. Sequencing statistics are provided in Table S2. A total of 592.46 million raw reads and 535.48 million high-quality reads were obtained, with more than 97% of Q20 values. On average, 88.63% of the reads were aligned to the reference genome of barley, and 85.06% of the reads were uniquely mapped (Table S3).

**Figure 1:**
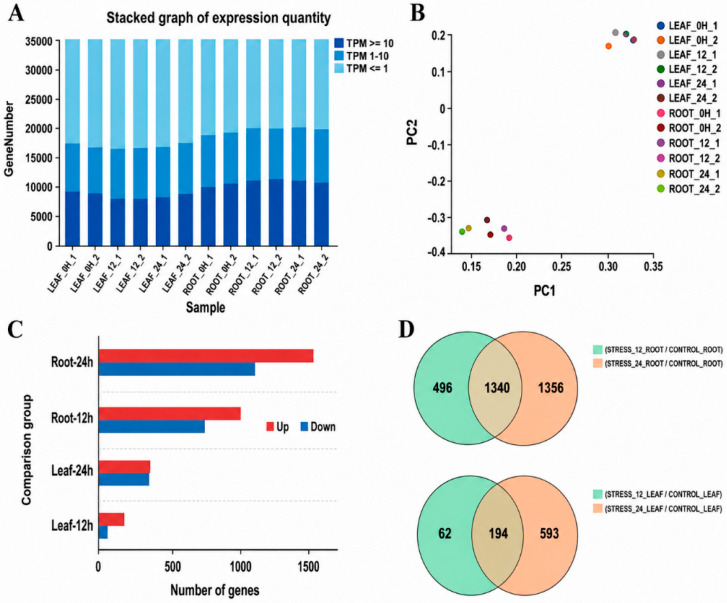
Analysis of barley line CC 89/Giza123 Transcriptome under 12 and 24 h salt stress of the root and leaf. (**A**) The expression of each gene (TPM) per sample: very low (<1); low (1–10); moderate to high (>10). (**B**) Principal component analysis (PCA) of the clustering of samples around PC1 and PC2. (**C**) The differentially expressed genes (DEGs) with varying levels of expression across groups, and the red bars and blue bars were used to represent up-regulation and down-regulation, respectively. (**D**) Venn diagram indicating overlapping of gene sets, where the numbers indicate common and exclusive genes.

Pearson’s correlation coefficients were used to measure the transcriptomic similarity between samples (Figure S1). Correlation values ranged from 0.5 to more than 0.8, with leaf-to-leaf comparisons showing the most agreement, while more variation was seen in root-to-root correlations (Figure S1). Gene expression levels were determined in Transcripts Per Million (TPM). Genes were classified into three expression classes: TPM < 1, TPM 1–10, and TPM > 10 (Figure 1A). Comprehensive TPM values are shown in Table S4. Principal Component Analysis (PCA) was used to determine the dominant pattern in the dataset. The first principal component separated samples primarily on the basis of tissue type (roots versus leaves) and highlighted tissue-specific expression as the dominant source of variation (Figure 1B).

### 3.2. Differential Gene Expression and Functional Annotation

Differential gene expression (DGE) analysis was carried out to identify transcripts that are salt stress responsive. Barley plants were treated with 200 mM NaCl for 12 h and 24 h, and each time point was compared to untreated controls. Pairwise comparisons were performed among each tissue and time point, as shown in Figure 1C. The differentially expressed genes (DEGs) were found to be different with respect to tissue type and stress duration. Leaves showed 256 DEGs at 12 h and 787 DEGs at 24 h, whereas roots showed 1836 and 2696 DEGs at 12 h and 24 h, respectively, suggesting a much higher transcriptional response in root tissue to salt exposure. A Venn diagram describing the overlapping and unique DEGs of roots and leaves among the various comparison groups and time points is presented in Figure 1D. Roots consisted of a larger DEG set than leaves. The intersection of the root DEGs between 12 h and 24 h contained 1340 genes, whereas 496 were unique to the 12 h sample, and 1356 were unique to the 24 h sample. For the leaves, 194 DEGs were shared, whereas 62 were specific to 12 h and 593 were specific to 24 h. These data support the conclusion that root tissues have a stronger and more dynamic transcriptional response to salinity than leaf tissues. Functional annotation of all the DEGs was carried out using a collection of databases, including NR, NT, GO, Swiss-Prot, InterPro, KEGG, and COG. Detailed annotation results of root and leaf samples at both time points are compiled in Supplementary Tables S5–S8.

### 3.3. GO Classification and Enrichment Analysis

Gene Ontology (GO) offers a hierarchical mechanism for describing the functions and locations of genes and is categorized into three groups: Biological Process (BP), Molecular Function (MF), and Cellular Component (CC). In order to investigate the functional roles of differentially expressed genes (DEGs) in response to salt stress, the GO annotation and enrichment analysis of the roots and leaves were conducted at 12 h and 24 h of salt stress treatment.

The BP terms of roots at 12 h salt stress were the biological regulation (157 genes), cellular processes (657), metabolic process (609), response to stimulus (178), and signaling (40). The terms related to MF were mainly dominated by binding (759), catalytic activity (760), and transcription regulator activity (35), and the terms that were related to CC included cell (697), organelle (489), and membrane (437) (Figure S2). The same patterns were observed at 24 h but with higher numbers of genes. The most represented BP terms included cellular process (952 genes), metabolic process (907), and response to stimulus (274). MF terms such as binding (1073) and catalytic activity (1108), and CC terms such as cell (1040) and membrane (672) also showed high representation (Figure S3).

The Gene Ontology (GO) classifications for differential expression analyses of leaves at 12 and 24 h of stress are given in Figure S4 and Figure S5, respectively. Figure S4 shows that the most important terms of biological process at 12 h stress were biological regulation (12), cellular process (89), metabolic process (97), regulation of biological process (10), response to stimulus (27), and signaling (3). Among the cellular component group, cell (78), cell part (78), organelle (38), extracellular region (29), and membrane part (77) were the predominant mapping sites of differentially expressed genes (DEGs). The most represented MF GO terms were binding (110), catalytic activity (147), transcription regulator activity (3), structural molecule activity (1), and transporter activity (9). Similarly, under 24 h stress, the terms biological regulation (67), cellular process (249), metabolic process (246), regulation of biological processes (61), response to stimulus (103), signaling (28), and multicellular organismal processes (30) were found most noted biological processes (Figure S5). In terms of cellular components, the DEGs were represented with high cell (233), cell part (233), supramolecular complex (8), organelle (120), membrane (284), and membrane part (255). For the molecular function, binding (334), catalytic activity (424), transcription regulator activity (15), structural molecule activity (10), and transporter activity (42) were the most represented GO terms.

### 3.4. GO Enrichment Analysis

**Figure 2:**
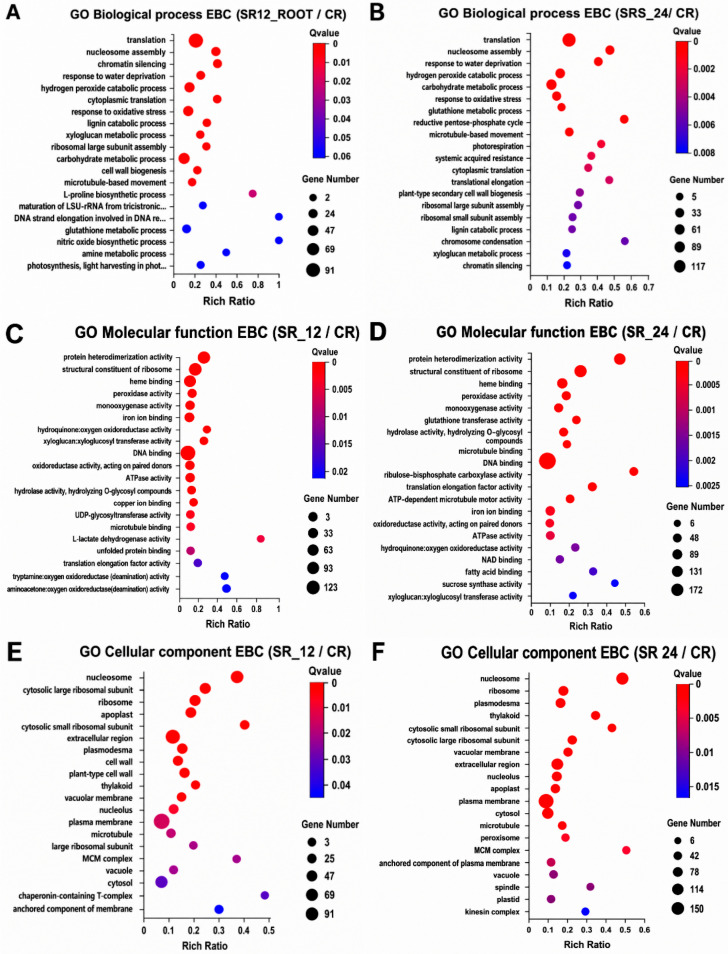
Gene ontology (GO) enrichment analysis of differentially expressed genes (DEGs) of roots under stress at 12 h and 24 h. The three GO categories analyzed are Biological Processes (BP), Molecular Functions (MF), and Cellular Components (CC). The following formats of the panels are (**A**) BP at 12 h; (**B**) BP at 24 h; (**C**) MF at 12 h; (**D**) MF at 24 h; (**E**) CC at 12 h; and (**F**) CC at 24 h. The *X*-axis indicates the ratio of enrichment of each GO term, which is defined as the percentage of DEGs annotated to a specific GO term in comparison with the percentage of all the genes in the genome annotated to the specific GO term. An increase in the enrichment ratios implies a higher specificity of the GO term to the DEGs. The bubble size is proportional to the number of DEGs relating to a particular GO term, which is an indicator of the prevalence of the term in the DEGs. The color of the bubbles represents the statistical significance of enrichment, with red showing low *p*-values and therefore higher confidence of the enrichment.

The phyper function of R was used to conduct enrichment analysis with *p*-values corrected with the Benjamini–Hochberg method to obtain Q-values. GO terms that had Q < 0.05 were significantly enriched. Figure 2A–F is a summary of enriched BP, MF, and CC terms of roots at 12 and 24 h of salt stress. Some of the enriched terms of BP that were common across tissues and time points were translation, nucleosome assembly, chromatin silencing, response to oxidative stress, response to water deprivation, and carbohydrate metabolic processes. MF terms protein heterodimerization activity, structural constituent of ribosome, heme binding, and monooxygenase activity were repeatedly enriched. CC terms were commonly related to nucleosome, ribosome, and thylakoid, which are important subcellular structures influenced by salt stress.

**Figure 3:**
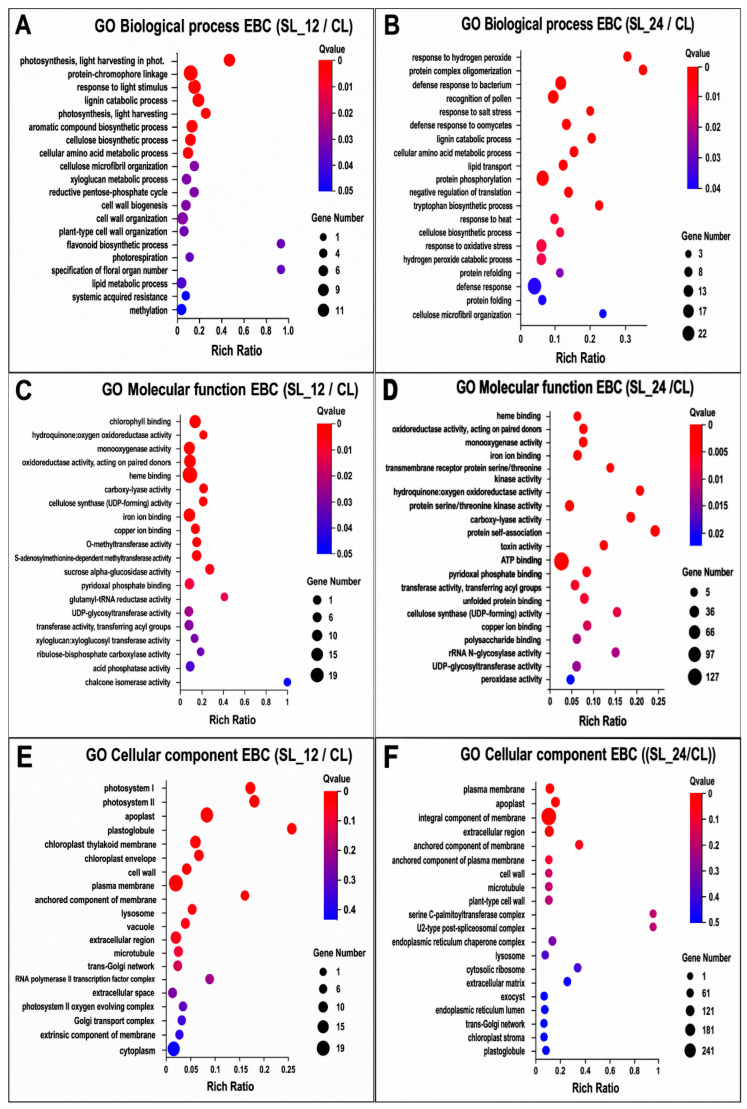
The enrichment analysis of differentially expressed genes (DEGs) in stressed (12 h and 24 h) leaves using Gene Ontology (GO). The three GO categories analyzed are Biological Processes (BP), Molecular Functions (MF), and Cellular Components (CC). The following panel formats are as follows: (**A**) BP at 12 h; (**B**) BP at 24 h; (**C**) MF at 12 h; (**D**) MF at 24 h; (**E**) CC at 12 h; and (**F**) CC at 24 h.

There were different functional profiles of leaves. GO terms, whose Q < 0.05 was found significant, were chosen to be enriched. Enriched BP, MF, and CC terms in leaves at 12 and 24 h of salt stress are summarized in Figure 3A–F. At 12 h, the enriched BP terms were connected with photosynthesis, light harvesting in photosystem I, nucleosome assembly, and response to light stimulus, which suggested functions in energy production and chromatin remodeling (Figure 3A–C). The 24 h shift in BP terms towards response to hydrogen peroxide, defense response to bacterium, and response to salt stress indicates the presence of oxidative stress and defense responses. MF leaf functions at 12 h were chlorophyll binding, heme binding, and monooxygenase, whereas those at 24 h involved ATP binding and protein serine/threonine kinase. At 12 h, photosystem I, photosystem II, and the thylakoid membrane of chloroplasts were associated with the CC terms, and at 24 h, the plasma membrane and extracellular region were enriched by the CC terms.

These results demonstrate tissue type and temporal functional responses to salt stress. Roots were highly enriched in both metabolic and regulatory processes, whereas leaves were highly enriched in both activities at an earlier stage of photosynthesis and later stages of the stress response pathways. Taken together, GO analysis underlines dynamic changes in molecules in barley in response to salt stress, which include energy metabolism, chromatin remodeling, oxidative stress reaction, and membrane-related processes.

### 3.5. Pathway Classification and Enrichment Analysis

To study the functional role of salt stress in barley roots and leaves, differentially expressed genes (DEGs) were mapped to KEGG pathways to identify five major functional groups: Metabolism, Genetic Information Processing, Environmental Information Processing, Organismal Systems, and Cellular Processes.

**Figure 4:**
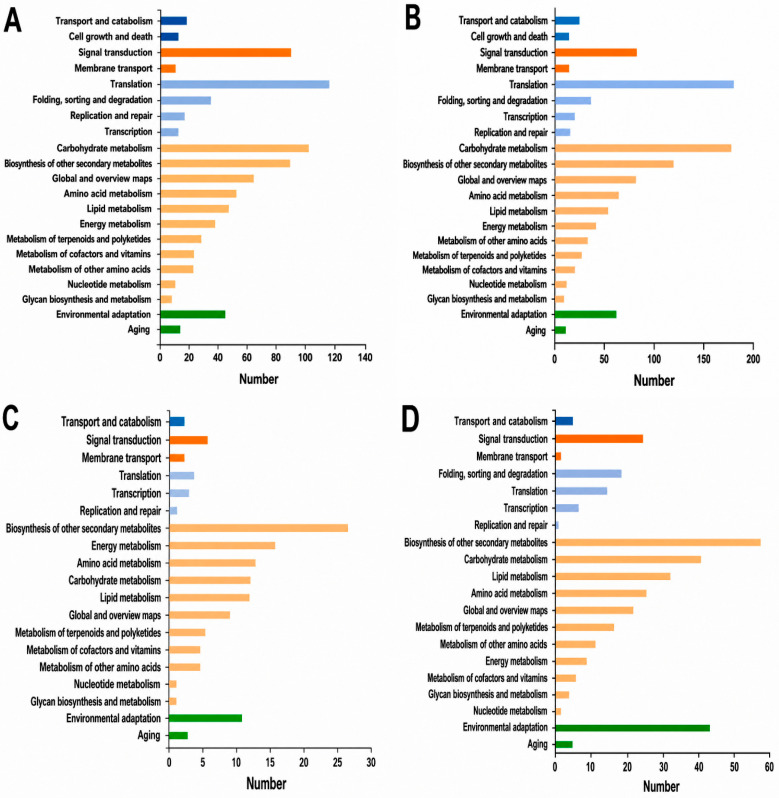
KEGG pathway analysis of genes of roots and leaves tissues exposed to salt stress for 12 h and 24 h. The figure shows how the annotated genes were distributed in both roots and leavessamples under salt stress in the categories of KEGG pathways. The classification of genes in the root tissue that has been subjected to the salt stresses is illustrated in Panel (**A**) (12 h) and Panel (**B**) (24 h). In the same way, Panel (**C**) shows the categorization of the genes in the leaves tissue following 12 h of salt stress, and Panel (**D**) shows the categorization of the genes in the leaf tissue following 24 h.

KEGG pathways were assigned to 792 DEGs, mapping to 135 pathways at 12 h in roots (Table S9; Figure 4A). The highest numbers were Metabolism (623 genes), Genetic Information Processing (221 genes), Environmental Information Processing (99 genes), Organismal Systems (104 genes), and Cellular Processes (46 genes). In Metabolism, biosynthesis of the secondary metabolites is highly represented (119 genes), and the process of genetic information processing is represented by translation (142 genes). Signal transduction of plant hormones (84 genes) and membrane transport (15 genes) were part of the environmental information processing. Environmental adaptation (60 genes) and aging (14 genes) were the organismal systems.

At 24 h, 1161 DEGs were mapped onto 138 KEGG pathways (Table S10; Figure 4B) in roots. The largest category was still Metabolism (715 genes), then Genetic Information Processing (322 genes), Environmental Information Processing (133 genes), Cellular Processes (76 genes), and Organismal Systems (103 genes). The major pathways were secondary metabolites biosynthesis (153 genes), translation (212 genes), and transport and catabolism (62 genes). Plant hormone signal transduction (119 genes) and membrane transport (14 genes) were once again observed, as well as the organismal systems, namely the environmental adaptation (87 genes) and aging (16 genes).

At 12 h, 122 DEGs in the leaf were mapped to 72 pathways (Table S11; Figure 4C). Most of them were associated with metabolism, specifically, the biosynthesis of secondary metabolites (32 genes). There were four translation-related genes in the processing of genetic information. Environmental information processing was characterized by the presence of plant hormone signal transduction (7 genes) and membrane transport (1 gene). Environmental adaptation (14 genes) and aging (2 genes) were found in organismal systems.

There were 358 DEGs at 24 h in leaves, which were associated with 110 KEGG pathways (Table S12; Figure 4D). The most common category was Metabolism, followed by genetic information processing and environmental information processing. The biosynthesis of secondary metabolites was the most represented, with 67 genes within Metabolism. The genetic information processing was involved in translation (21 genes). The information processing that was related to the environment was divided into plant hormone signal transduction (33 genes) and membrane transport (4 genes). Other organismal systems were environmental adaptation (68 genes) and aging (8 genes).

### 3.6. KEGG Pathway Enrichment

**Figure 5:**
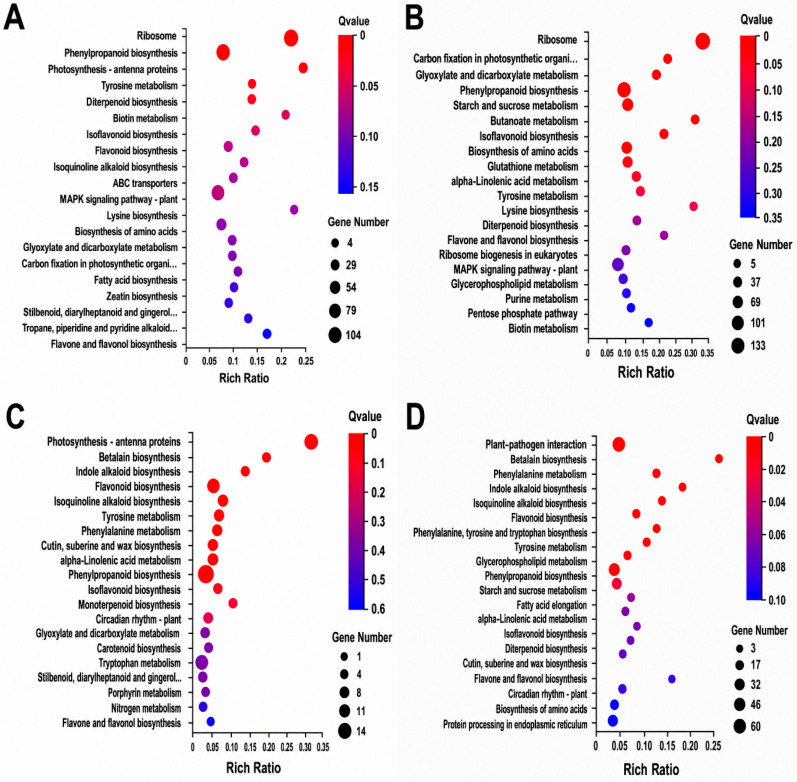
Enriched KEGG pathways of differentially expressed genes (DEGs) of roots and leaves after salt treatment at 12 and 24 h. Panels (**A**,**B**) illustrate the enriched KEGG pathways of the DEGs in roots at 12 h and 24 h of salt stress, respectively. Similarly, Panels (**C**,**D**) illustrate the enriched KEGG pathways of the DEGs in leaves at 12 h and 24 h after salt stress, respectively.

In the roots, at 12 h, the ribosome pathway showed the highest enrichment, indicating rapid modulation of the protein synthesis machinery. Several secondary metabolite biosynthesis pathways, including phenylpropanoid, flavonoid, isoquinoline alkaloid, and diterpenoid biosynthesis, were also significantly enriched, suggesting activation of stress-responsive metabolic processes. Pathways related to photosynthesis-antenna proteins and tyrosine metabolism also appeared among the enriched categories, reflecting early signaling and metabolic reprogramming (Figure 5A). However, at 24 h of salt stress, the enrichment pattern shifted toward pathways associated with sustained metabolic adaptation. Carbon fixation in photosynthetic organisms, glyoxylate and dicarboxylate metabolism, and photosynthesis were among the most enriched pathways, indicating deeper metabolic restructuring under prolonged stress. The ribosome pathway remained significantly enriched, though with altered gene representation compared to 12 h. Secondary metabolite pathways such as phenylpropanoid biosynthesis continued to be enriched, highlighting ongoing reinforcement of stress-related metabolic defenses (Figure 5B).

Similarly, in leaves at 12 h, the enriched pathways related to photosynthesis, particularly photosynthesis-antenna proteins, reflect rapid adjustments in light-harvesting processes. Several specialized metabolic pathways, including betalain, indole alkaloid, flavonoid, and isoquinoline alkaloid biosynthesis, were also enriched, indicating activation of protective secondary metabolites. The overall number of enriched pathways was lower than in roots, suggesting a more targeted early response in leaf tissues (Figure 5C). Similarly, at 24 h, leaves exhibited a broader enrichment profile. The plant pathogen interaction pathway became prominently enriched, implying activation of defense signaling cascades under prolonged salt exposure. Secondary metabolite pathways such as betalain, indole alkaloid, and isoquinoline alkaloid biosynthesis remained enriched, consistent with continued accumulation of protective compounds. Pathways related to amino acid biosynthesis (e.g., phenylalanine, tyrosine, and tryptophan biosynthesis) were also enriched, indicating metabolic adjustments supporting stress tolerance (Figure 5D).

### 3.7. Regulatory and Functional DEGs in Roots and Leaves

This research identified a number of potential salt-responsive genes that were differentially expressed in both roots and leaves at 12 h and 24 h of salt stress, which have been previously confirmed in plants. Hence, Table 1 lists key candidate genes specifically associated with salt tolerance in barley and is categorized into two broad groups: regulatory and functional genes, with emphasis on tissue-specific and time-dependent responses. Supplementary Tables S5–S8 present the same data, along with the statistical significance of the expression differences (*p*-values).

#### 3.7.1. Regulatory DEGs

The regulatory genes were also highly represented in roots; at 12 h, 40 mitogen-activated protein kinases were up-regulated and 16 were down-regulated; at 24 h, 49 genes were up-regulated, and 24 genes were down-regulated. The serine/threonine-protein kinases showed strong down-regulation (79 genes at 12 h and 87 genes at 24 h), although some genes were also up-regulated. The histidine kinase domain was detected exclusively after 12 h, while protein phosphatase 2C (PP2C), late embryogenesis abundant (LEA) proteins, and other phosphatases were continually up-regulated. The other regulatory elements, such as phospholipases and calmodulin, showed largely up-regulation at both time points.

In leaves, mitogen-activated protein kinase kinase has moderate expression at 12 h (3 genes up-regulated, 3 genes down-regulated) and higher at 24 h (7 genes up-regulated, 16 genes down-regulated). The down-regulation of serine/threonine-protein kinases was very high (14 genes at 12 h and 81 genes at 24 h), and protein phosphatase 2C (PP2C) and protein-serine/threonine phosphatases were consistently up-regulated. Proteins containing leucine-rich repeats were largely down-regulated, and phospholipases, with a majority of proteins, were up-regulated at 12 h and 24 h of salt stress. Calmodulin genes were mostly down-regulated at 24 h.

The patterns of transcription factors (TFs) were different across both tissues. WRKY and basic helix-loop-helix TFs in roots were differentially expressed, with modest changes at 12 h and increased activity at 24 h, whereas heat shock TFs were up-regulated at these two time points. In leaves, WRKY TFs were mainly down-regulated at 24 h of salt stress, while heat shock TFs also showed down-regulation under prolonged stress.

#### 3.7.2. Functional DEGs

The salt stress induced the antioxidant defenses in both tissues. In roots, Peroxidases were highly up-regulated (23 genes at 12 h, 30 genes at 24 h), as well as catalase, ascorbate peroxidase, and glutathione peroxidase were also up-regulated. Peroxidases were also prominent in leaves (5 genes at 12 h and 8 genes at 24 h), and further up-regulation of catalase and ascorbate peroxidase was also observed at 24 h. Glutathione S-transferase and metallothionein showed down-regulation.

Defensive stress proteins like late embryogenesis abundant proteins and dehydrins were highly up-regulated in roots, especially at 24 h, suggesting functions of osmotic adjustment. The pathogenesis-related proteins were exclusively up-regulated. The same tendencies were observed in leaves, in which the proteins of heat shock and dehydrins were most expressed at 24 h. The pathogenesis-related proteins were also differentially expressed, showing down-regulation at 24 h.

In the transport and Channels category, the up-regulation of aquaporins (MIP and TIP types) in roots and leaves at the two time points facilitated water movement during osmotic stress. At 12 h, ABC transporters were substantially up-regulated in roots, and at 24 h, they remained active, while the leaves also showed up-regulation. Ion transporters, such as potassium and calcium channels, also exhibited changing patterns, and some were down-regulated in later stages.

In the metabolic category, there was a large up-regulation of genes encoding the cytochrome P450 superfamily in both tissues, roots (40 genes at 12 h and 50 genes at 24 h) and leaves (11 genes at 12 h and 15 genes at 24 h). The binding proteins of chlorophyll a and b were significantly increased in the leaves, indicating changes in the photosynthetic process. Other components, such as flavoproteins and ferredoxins, were observed at low frequencies.

The identification of regulatory and functional DEGs in roots and leaves supports the complexity of the adaptive response of barley to salinity stress. Roots seem to develop a more effective regulatory response, and leaves show dynamic responses in photosynthetic and signaling pathways. The results obtained can be invaluable in understanding the molecular basis of salt-tolerant and identifying the candidate genes to be used in crop-enhancing programs in the future.

### 3.8. Validation of RNA-Seq Data by Quantitative Real-Time PCR

**Figure 6:**
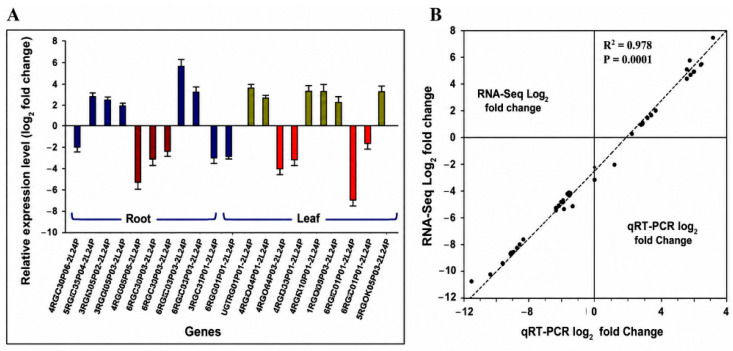
(**A**) Quantitative PCR validation of transcription data depicting fold-change variation of selected salinity-tolerance genes across roots and leaves tissues. (**B**) Scatter plot showing correlation between RNA-Seq and RT-PCR expression values measured by RNA sequencing (*y*-axis, log_2_-transformed) and quantitative RT-PCR (*x*-axis, log_2_-transformed). Each dot represents an individual gene. The strong linear distribution along the diagonal indicates a high positive correlation between the two methods.

In order to confirm the transcriptomic patterns which have been observed in the analysis of the RNA-sequences, a total of 21 genes (9 genes represent root transcriptome and 12 genes represent leaf transcriptome) were chosen for quantitative reverse transcription PCR (qRT-PCR) validation. These genes were tested in the presence of 12 h and 24 h salt stress conditions, showing the patterns of fold-change in the expression of the genes (Figure 6A) in response to 12- and 24-h salt stress conditions. The correlation analysis further demonstrated a strong positive relationship between log_2_-transformed RNA-Seq and qRT-PCR expression values, indicating excellent agreement between the two methods. This implies that RNA-Seq quantification accurately reflects true gene expression, as independently verified by qRT-PCR (Figure 6B).

## 4. Discussion

### 4.1. Characterization of Transcriptome Under Salt Stress

The present investigation aimed to explain transcriptomic responses of salinity tolerant barley line CC89/Giza123 in the roots and leaves under salinity stress with the purpose of identifying the candidate genes related to salt tolerance. Using the Illumina HiSeq 4000 sequencing platform, twelve cDNA libraries were sequenced, producing a cumulative number of 592.46 million raw reads, and 535.48 million high-quality clean reads were obtained. Each library had an average of 49.37 and 44.62 million raw and clean reads, respectively. Alignment against the reference genome resulted in an average mapping rate of 88.63 per cent and therefore enabled complete coverage for subsequent analyses. Differential gene expression analysis showed the presence of a significant number of differentially expressed genes (DEGs) in both roots and leaves at 12 and 24 h after exposure to 200 mM NaCl (Figure 2). Notably, the number of DEGs was found to be higher at 24 h than at 12 h, and roots were consistently found to have a higher DEG number than leaves. These observations are consistent with the previous studies on barley in which salt stress accumulation resulted in an increase in transcriptional activity [33,34]. The higher number of DEG at 24 h indicates that this time point is ideal for recording the full range of stress response genes. Interestingly, a contrasting trend was reported by Bahieldin et al. [35], i.e., a decrease in DEG numbers at 24 h compared to 12 h under severe salt stress (400 mM NaCl). This discrepancy is potentially due to the differences in concentration of salt, as the present study has used a moderate level of stress (200 mM NaCl), which might provide a more sustained transcriptional response. In this study, roots exhibited a significantly stronger transcriptional response than leaves at both time points, reflecting their main role in sensing and responding to soil salinity. At 24 h, more than three times as many DEGs were found in roots compared to leaves, whereas at 12 h, root DEGs were found to be seven times more abundant than leaf DEGs. This early and strong activation in roots highlights their key role in driving the initiation of adaptive responses to salinity. These observations agree with findings in other species. For example, Baldoni et al. [36] reported that in a salt-tolerant rice genotype (Eurosis), roots presented more DEGs than leaves at 3 h stress duration, with more up-regulated (61.6%) than down-regulated (38.4%). Similarly, Luo et al. [37] reported that sweet potato roots had more DEGs overall and a higher proportion of up-regulated genes with comparison to sweet potato leaves that showed fewer DEGs and were found to have predominance of down-regulation. The general finding of these results is that barley is a tissue-specific, time-dependent response to salt stress, and roots are very crucial in the early detection and response to salt stress. The entire dataset of transcriptome reports obtained in the current study is an excellent source of information on the discovery of major regulatory and functional genes in salt tolerance. Such genes can also be potential targets of genetic enhancement in barley and other crop cereals.

### 4.2. Functional Characterization of Differential Expression

The functional analysis of differentially expressed genes (DEGs) is a crucial part of transcriptomic studies and offers information about the biological processes and molecular pathways that are activated in response to environmental stimuli. Gene Ontology (GO) annotation and Kyoto Encyclopedia of Genes and Genomes (KEGG) pathway analysis are extremely popular tools for the functional interpretation of gene expression data. In this study, GO and KEGG pathway analyses present the tissue- and time-dependent functional responses of barley to salt stress. Roots had a greater and more extensive transcriptional reaction than leaves, especially at 24 h, which shows that they are the main receivers of salt perception and early adaptive signaling. The increase in biological processes associated with cellular and metabolic processes, responses to stimuli, and signaling in roots indicates rapid activation of stress-sensitive regulatory networks. Such comprehensive metabolic and regulation changes in salinity have been observed in barley and other cereals [38,39,40].

GO enrichment pattern invariably emphasized translation, assembling nucleosomes, silencing of chromatin, and response to oxidative stresses across tissues and time points. The dramatic enhancement of ribosome-associated molecular functions and structural elements implies the active remodeling of protein synthesis machineries, a characteristic feature of the initial stress response [41]. The enrichment of terms related to chromatin enrichment promotes the idea that epigenetic regulation is a component of salt-induced transcriptional control, which is in line with the emerging data on the involvement of chromatin remodeling in abiotic stress tolerance [42,43,44].

Photosynthesis-related processes (especially those of photosystem I and II and light-harvesting complexes) in leaf tissues were early enriched, especially at 12 h. This is probably a fast adjustment of the energy metabolism to counteract osmotic imbalance. Nonetheless, after 24 h, the enrichment trend changed to oxidative stress, hydrogen peroxide response, and defense-associated pathways, which showed progressive damage and activation of protective mechanisms induced by stress. This shift in photosynthetic adaptation to defense signaling is in line with the earlier studies detailing salt-induced oxidative stress and the presence of ROS in triggering signaling by ROS in leaves [45,46].

The analysis of the KEGG pathways also supported these results. Rapid activation of protein synthesis and metabolic stress-related defenses is seen by strong enrichment of ribosome, secondary metabolite biosynthesis (phenylpropanoid, flavonoid, diterpenoid), and hormone signal transduction pathways in roots at 12 h. The secondary metabolites are highly, especially phenylpropanoids and flavonoids, which are antioxidant and protective when it comes to salinity [38,47,48]. Enrichment of carbon fixation, glyoxylate and dicarboxylate metabolism, and photosynthesis-related pathways at 24 h are indicative of greater metabolite restructuring in response to long-term exposure to stress. This continued enrichment of the plant hormone signal transduction pathways also demonstrates the significance of hormonal crosstalk in salt tolerance processes [23].

KEGG patterns of enrichment in leaves were found to be more highly focused at 12 h, predominantly on photosynthesis-antenna proteins and specialized biosynthesis of metabolites. After 24 h, more defense-related pathways like plant-pathogen interaction and amino acid biosynthesis became enriched, which is an indication of activating systemic stress response processes [48].

In general, both combined GO and KEGG studies have shown dynamic and tissue-specific molecular responses in barley to salt stress. Roots are primary sensors and regulators, being regulated by widespread changes in metabolic and translational reprogramming, while leaves first change photosynthetic activity and then induce oxidative and defense reactions. These are important findings that help to understand the coordinated regulatory networks that are the basis of salinity tolerance and provide some targets for genetic enhancement of barley and other cereal crops.

### 4.3. Characterization of Potential Salt-Responsive DEGs

This study aimed at identifying and functional characterization of salt-responsive genes in the barley line CC89/Giza123 with interests on understanding the effects of salinity stress on physiological, metabolic, and cellular processes. Transcriptomic analysis showed a wide range of differentially expressed genes (DEGs) in roots and leaves at 12 h and 24 h of salt stress. These DEGs were divided into two major categories, which include the regulatory genes and functional genes (Table 1).

### 4.4. Regulatory Genes: Signal Transduction and Transcriptional Regulation

Regulatory DEGs were involved with transcription factors (TFs), protein kinases, phosphatases, and hormone signaling enzymes. Of the various signaling pathways, protein kinases and phosphatases are key players in cellular signaling that involve the transduction of salt stress signals into transcriptional and metabolic responses [49,50]. Salt stress affected the expression and activity of these regulators, especially in roots, where mitogen-activated protein kinase-containing genes were mostly up-regulated at both 12 and 24 h. In contrast, leaves showed a general down-regulation of these genes, though several were also up-regulated (Table 1). These results are in agreement with those of other studies [35,51,52], strengthening the idea that roots act as main sensors and regulators of salt stress.

Early Effectors of Stress Response Transcription factors are a pivotal element in the regulation of stress-responsive gene expression. DEGs encoding TFs like bHLH, WRKY, and heat shock transcription factors were differentially expressed in both tissues, with balanced regulation in roots (Table 1). Similar TF families have been found in salt-stressed barley mutants [18]. TFs are usually early induced genes by upstream kinases and phosphatases [53]. Notably, bHLH genes were generally down-regulated, although specific family members, such as bHLH122, were induced in agreement with its role in enhancing levels of ABA by repressing ABA catabolism in Arabidopsis [54].

### 4.5. Functional Genes: Osmotic Adjustment, Detoxification and Stress Protection

Salt stress induces both osmotic and ionic stress, leading to water deficit and ion imbalance. Plants counteract these effects through mechanisms such as ion transport, osmolyte production, and the activation of stress-responsive proteins. Ion channels are important for maintaining ionic equilibrium, membrane stability, and signal transduction. DEGs for aquaporins and other important water channel proteins were up-regulated, especially in roots, supporting their function in maintaining hydration and photosynthetic activity under stress [55]. ATP-binding cassette (ABC) transporters have been reported to be involved in stomatal closure and stress regulation and were up-regulated in both roots and leaves (Table 1), supporting their role in abiotic stress adaptation [56]. Reactive oxygen species (ROS) buildup under salt stress requires efficient antioxidant enzymes. DEGs encoding catalase, peroxidase, and ascorbate peroxidase were highly expressed in both tissues, indicating activation of detoxification pathways. These results agree with the earlier reports in barley and other crops [33,35,57,58,59].

Late embryogenesis abundant (LEA) proteins and dehydrins (DHNs) were largely highly up-regulated in both tissues. These hydrophilic proteins help safeguard the cells from dehydration and oxidative damage as antioxidants, ion sequestrants and stabilizers of the cellular structures [60,61,62,63,64]. Their high expression highlights the significance they have in providing salt stress tolerance. Heat shock proteins (HSPs) as molecular chaperones were expressed differently as well, with heat shock factors (HSFs) being more highly expressed in leaves. HSFs are responsible for HSPs regulation and for general stress responses such as water deficiency and non-thermal stresses [65,66].

### 4.6. Remodeling and Regulation of Cell-Wall Growth

High intracellular levels of Na^+^ and Cl^-^ can affect cell division and expansion, thus reducing germination and seed viability. Several differentially expressed genes (DEGs) involved in cell-wall loosening, e.g., xyloglucan endotransglucosylase/hydrolase, chitinase, and expansin, were differentially expressed in response to salt stress (Tables S5–S8). The expression of xyloglucan endotransglucosylase and chitinase was up- and down-regulated in roots and leaves, respectively, while expansin showed the opposite pattern. Expansin proteins initiate cell expansion by hydrogen bond disruption between cellulose and xyloglucan, and their up-regulation under saline stress has been previously reported in maize [67,68]. These results suggest that cell-wall remodeling is involved in salt tolerance through modulation of growth dynamics.

Overall, this study identified a large number of salt-responsive genes in the barley line CC89/Giza123, which were grouped into regulatory and functional groups. Regulatory DEGs comprised transcription factors, protein kinases, phosphatases, and hormone signaling components, with roots exhibiting higher and earlier activation than leaves. Protein kinases and phosphatases were mainly up-regulated in the roots, suggesting active signal transduction. Transcription factors like WRKY, bHLH, and HSFs were differentially expressed, with roots exhibiting more up-regulation. Functional DEGs were involved in osmotic adjustment, ion transport, antioxidant defense, and protection against stress. Aquaporins, ABC transporters, and ion channels were also up-regulated, and this helps transport water and maintain ion homeostasis. Antioxidant enzymes (e.g., catalase, peroxidase) were highly expressed in order to reduce the ROS damage. LEA proteins and dehydrins were highly induced because they protect the cells from dehydration. Metabolic genes involved in carbohydrate, fatty acid, and secondary metabolite biosynthesis were variably expressed, which represents energy reprogramming under stress. Cell-wall remodeling genes (e.g., expansin, chitinase) were tissue-specific, supporting growth adaptation. These results point to the coordinated molecular response of barley to salinity, with the roots being major sensors and regulators and candidate genes for salt tolerance improvement in crops.

## 5. Conclusions

This study presents a detailed analysis of the gene expression profile in roots and leaves of a salt-tolerant barley line CC89/Giza123 under acute salinity stress (200 mM NaCl) at 12 h and 24 h time points. RNA-sequencing-based differential expression analysis showed a large number of significantly regulated genes for all the treatment combinations. Roots and leaves use different strategies to cope with salt stress, and these strategies change over time. Roots respond quickly by adjusting protein synthesis and activating protective metabolites, then shift toward long-term metabolic adjustments as stress continues. Leaves begin with targeted changes in photosynthesis and later broaden their defense and metabolic responses. The transcriptional response was defined by the early activation of genes involved in hormone signaling, kinase-mediated signaling, and phosphatase activity, pointing to the centrality of signal transduction in the early perception of stress. Transcription factors, especially WRKY and bHLH family members, were well represented among the differentially expressed genes, highlighting their role in regulating salinity tolerance. Also, the work has identified dynamic regulation of genes associated with antioxidant response, osmolyte synthesis, and membrane transport, providing evidence of a complex molecular response to counteract salt-induced stress. Together, these findings show that salt tolerance relies on coordinated but distinct responses in different plant tissues. Understanding these patterns can guide future efforts to improve plant resilience under salinity. These salt-responsive candidate genes can serve as useful genetic material in the future for functional validation and breeding schemes to enhance salinity tolerance in barley and other cereal crops.

## Figures and Tables

**Table 1:** Categorization of salinity-responsive differentially expressed genes in roots and leaves (under 12 h and 24 h stress conditions) based on sequence similarity to previously characterized genes.

Category	Root-12h-Up	Root-12h-Down	Root-24h-Up	Root-24h-Down	Leaf-12h-Up	Leaf-12h-Down	Leaf-24h-Up	Leaf-24h-Down
**Protein Kinases and Phosphatases**								
Histidine kinase domain	2	0	0	0	–	–	–	–
Mitogen-activated protein kinase	40	16	49	24	3	3	7	16
Serine/threonine-protein kinase	13	79	28	87	2	14	7	81
Protein phosphatase 2C	7	2	3	2	1	0	3	0
Protein-serine/threonine phosphatase	–	–	–	–	1	0	3	0
Phospholipase	8	3	8	6	3	0	6	4
Calmodulin	5	0	6	1	0	1	1	6
Leucine-rich repeats	2	0	1	0	2	4	3	23
**Transcription Factors (TFs)**								
WRKY DNA-binding protein	1	1	2	2	0	1	0	10
Basic helix-loop-helix TF	4	5	8	7	1	0	1	1
Heat shock TF C1	2	0	2	0	0	1	0	1
LRRNT_2 domain protein	–	–	–	–	0	2	0	4
**Antioxidants and Detoxifying Enzymes**								
Peroxidase	23	9	30	16	5	0	8	7
Catalase	1	0	1	0	0	0	1	0
Ascorbate peroxidase	1	0	1	0	0	0	1	0
Glutathione peroxidase	0	0	1	0	0	0	0	0
Glutathione S-transferase	1	2	0	0	0	1	0	4
Metallothionein	2	0	2	0	0	0	0	1
**Defensive Proteins**								
Late embryogenesis abundant protein	3	1	10	1	1	0	2	3
Dehydrin	4	0	3	0	0	0	3	0
Pathogenesis-related protein	9	0	11	0	1	0	1	6
Heat shock protein	1	3	3	5	1	0	9	0
**Channels and Transporters**								
Aquaporin	3	1	4	2	1	0	1	0
ABC transporter	16	0	12	0	3	0	5	5
Potassium transporters	4	2	1	5	0	0	1	2
Calcium channel/exchanger	3	0	1	1	–	–	–	–
Ligand-gated ion channel	–	–	–	–	0	1	0	2
**Stress-Responsive Metabolic Proteins**								
Cytochrome P450 superfamily	40	4	50	7	11	3	15	11
Flavoproteins	2	1	3	4	0	0	0	1
Ferredoxins	–	–	–	–	1	0	0	2
Chlorophyll a-b binding protein	–	–	–	–	11	0	7	0

## Data Availability

The original contributions presented in this study are included in the article’s Supplementary Material. Further inquiries can be directed to the corresponding author.

## Supplementary Materials

The following supporting information can be downloaded at https://www.mdpi.com/article/10.3390/cimb48070718/s1.
