# Supplementary material for: Comparative Transcriptome Analysis of Leaves and Roots Responses in Salt-Tolerant Barley Line CC89/Giza123 Under Salinity Stress
Source: Curr Issues Mol Biol. 2026 Jul 14;48(7):718. doi: 10.3390/cimb48070718 (PMC13407700; doi:10.3390/cimb48070718)
Supplement: Supplementary file 1 [file cimb-48-00718-s001.zip › cimb-4345891-supplementary-7.8/Supplementary files.pdf]

Supplementary Files

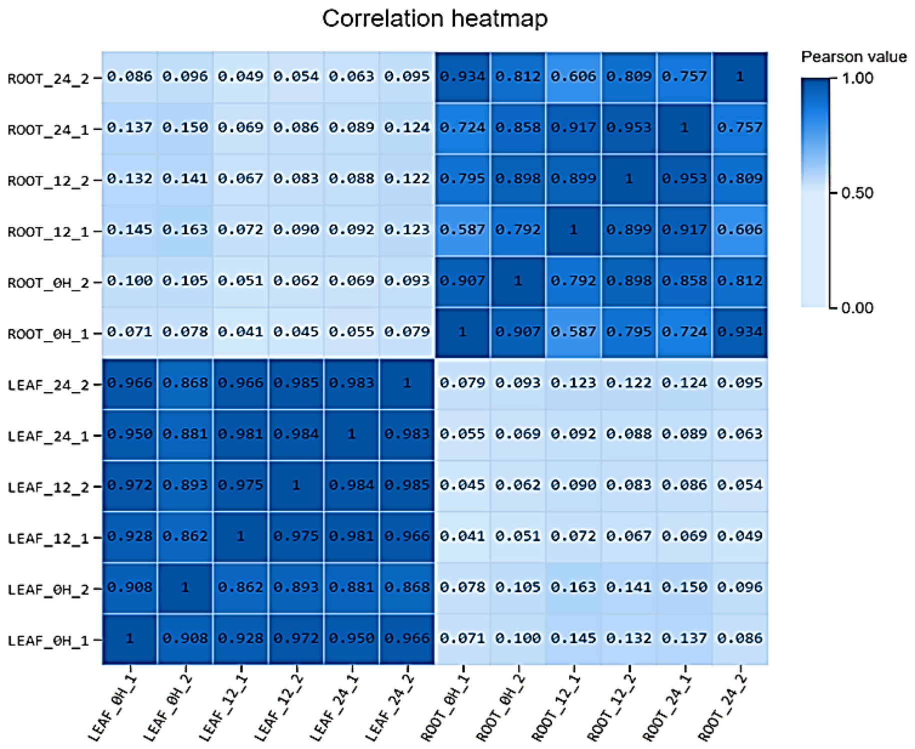

Figure S1. Correlation matrix of sample relationships. This figure presents a correlation matrix plot illustrating the pairwise Pearson correlation coefficients among the analyzed samples. Both the X and Y axes represent distinct sample names. The color intensity of each cell reflects the strength of correlation between sample pairs, with darker color indicating stronger positive correlations and lighter color denoting weaker associations.

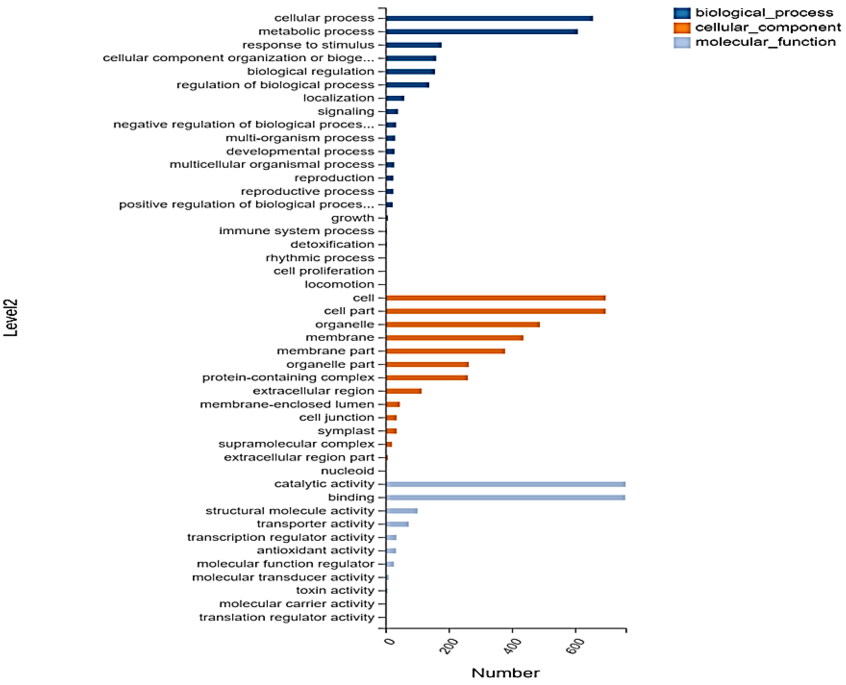

Figure S2. Gene Ontology (GO) functional categorization in CONTROL_ROOT and STRESS_12_ROOT samples. This figure illustrates the distribution of genes across GO terms in CONTROL_ROOT and STRESS_12_ROOT conditions. The X-axis represents the number of genes assigned to each GO term, while the Y-axis groups the GO terms based on their functional roles, enabling comparison of biological processes, molecular functions, and cellular components between the two sample sets.

https://doi.org/10.3390/xxxxx

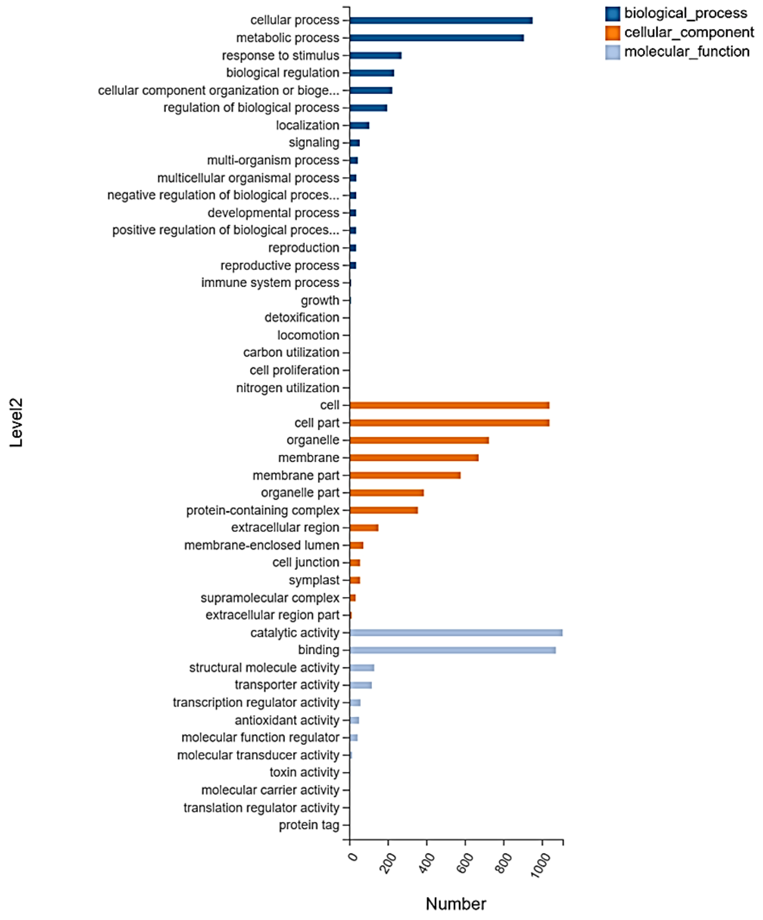

Figure S3. GO functional classification of genes in CONTROL_ROOT and STRESS_24_ROOT samples. This figure depicts the distribution of genes across Gene Ontology (GO) terms under CONTROL_ROOT and STRESS_24_ROOT conditions. The X-axis indicates the number of genes associated with each GO term, while the Y-axis organizes the GO terms according to their functional categories, including biological processes, molecular functions, and cellular components.

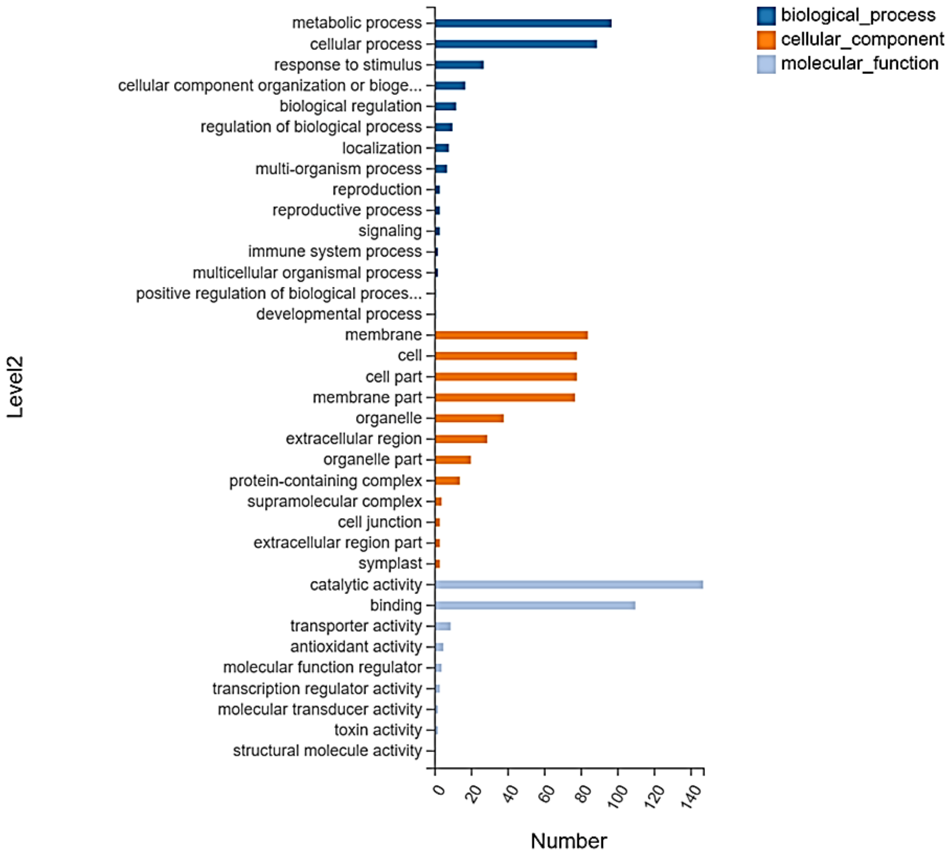

Figure S4. GO functional categorization of genes in leaf tissue under 12-hour salt stress. This graph illustrates the distribution of genes across Gene Ontology (GO) terms in leaf samples subjected to 12 hours of salt stress. The X-axis represents the number of genes assigned to each GO term, while the Y-axis displays the corresponding GO functional categories, including biological processes, molecular functions, and cellular components.

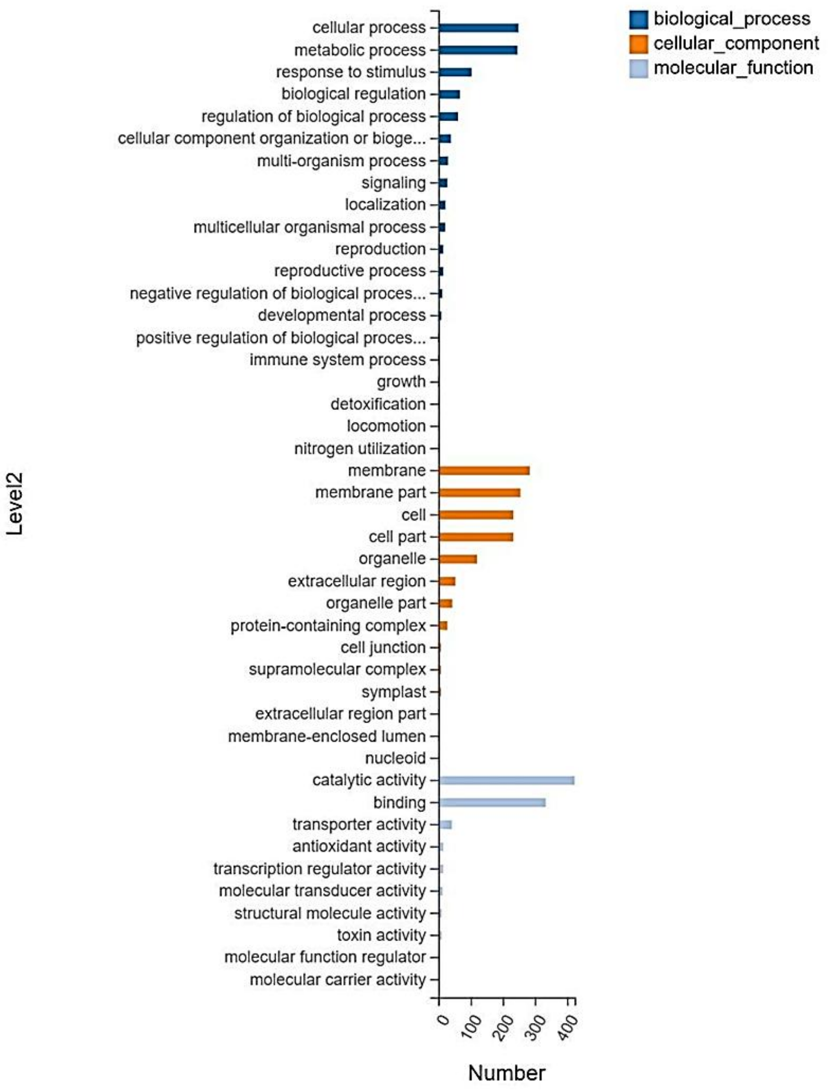

Figure S5. GO functional categorization of genes in leaf tissue under 24-hour salt stress. This graph illustrates the distribution of genes across Gene Ontology (GO) terms in leaf samples subjected to 12 hours of salt stress. The X-axis represents the number of genes assigned to each GO term, while the Y-axis displays the corresponding GO functional categories, including biological processes, molecular functions, and cellular components.

https://doi.org/10.3390/xxxxx

Table S1. Gene-Specific Primers Used for Quantitative PCR Validation of RNA-Seq Results.

Primer name	*TM(°C)*	*Forward Sequence*	*Reverse Sequence*	*Prod Size*
1HG0060530-LC12D	55.33	ACCAAACACTACTTTGGAGGT	CCATTTCAAAGACCTCATTC	176
6HG0522520-LC12D	55.1	TATGTATGGAAAGGCTCAAAA	TTCACTAGCACAACTGAGGAT	182
5HG0364210-LC12UP	54.58	TAAATCCAAACCTCACATCAT	AGAACGTCTCGTGGATGTAG	153
UnG0625590-LC12UP	54.92	CACAAGTTCTTGTCCTTCAAC	CACTGTGAGACCATTTTTCAT	180
4HG0276680-LC24D	55.09	GGAGCATTTTTCAGGATTATT	TAAGCGTATCAATGTTGGTCT	182
1HG0060530-LC24D	55.33	ACCAAACACTACTTTGGAGGT	CCATTTCAAAGACCTCATTC	176
4HG0314970-LC24D	54.68	CAGAAGGAAATTCAAAACTCA	GGACTATGATCATTGGGGTAT	184
2HG0111410-LC24UP	55.24	AAGGAGAAGCACCACAGAC	GTCCTGCAAGTAGAAGGAAG	153
1HG0073360-LC24UP	54.61	GTCCAACTGGATGGGTTAT	CTGTACTCGTCGTGAGCATA	180
7HG0570260-LC24UP	54.91	AGATGACCAAGACCAACAAC	GACAGCAGGTCGAACGTAT	190
6HG0523140-L24-12D	55.32	CATATCTGACGAAATGAAGGA	AGGTAGATTTGTTGAGCATGA	179
2HG0135750-L24-12D	55.5	ATCCTCTACACCCTCATCATC	TGCCTCTATATGGTAATTGGA	183
3HG0197310-L24-12UP	55.6	AGGGAGGAGAAGGAGGAC	GATGGTCTTGGGCTTCTTA	183
4HG0324450-RC-12D	55.42	GGCTATAGTTCCACTTCCATC	ATGAGAGTGTCATCGCAGA	176
5HG0413180-RC12UP	54.8	AAGGTCAACTTCCCCAAC	GAAGCTCATGTAGCTCTCGTA	198
1HG0048510-RC12UP	55.12	CCTGCTAATGATCCTCCTTAT	GTTGCTGGAGTCGAACAC	208
3HG0235080-RC12UP	55.06	TTCATCTTCATCAGGGAGAC	TAGAGCATGACGAGGAAGTAG	173
4HG0340080-RC24D	55.17	AACAAGGAGATCTCAGAGGAG	GAAGATGTCCTTCTGGAACA	177
6HG0460220-RC24D	56.76	CAAAGCATCCTAGCTATTTGG	ATTCTTGATCTCCTTCACCTT	186
4HG0291920-RC24D	54.86	TTCTTCATATGGTCTCCTCAA	AATCCTCTCACCACAATAGGT	180
6HG0521410-RC24UP	55.29	ACTACGACTTCTGCAAGAACA	GCTGCTGAAGTTGAGAGACT	164
2HG0162330-RC24UP	54.51	GCTTGCTGTGGTGGTACT	TCTTCATGTTCTTACACACGA	140

Table S2. Quality Statistics of Transcriptome Sequencing Reads Across Twelve Barley RNA Libraries.

Sample	Total Raw Reads (M)	Total Clean Reads (M)	Total Clean Bases (Gb)	Clean Reads Q20 (%)	Clean Reads Q30 (%)	Clean Reads Ratio (%)
LEAF_0H_1	49.08	45.18	6.78	97.45	89.56	92.06
LEAF_0H_2	49.08	44.89	6.73	97.66	90.31	91.47
LEAF_12_1	49.08	43.96	6.59	97.5	89.81	89.57
LEAF_12_2	49.08	44.42	6.66	97.5	89.8	90.5
LEAF_24_1	49.08	44.39	6.66	97.35	89.28	90.44
LEAF_24_2	49.08	44.48	6.67	97.19	88.78	90.63

ROOT_0H_1	50.83	45.24	6.79	97.51	89.81	89
ROOT_0H_2	49.08	43.83	6.57	97.67	90.34	89.31
ROOT_12_1	50.83	45.35	6.8	97.53	89.91	89.22
ROOT_12_2	49.08	44.61	6.69	97.52	89.8	90.89
ROOT_24_1	49.08	44.35	6.65	97.66	90.28	90.35
ROOT_24_2	49.08	44.78	6.72	97.44	89.52	91.24

https://doi.org/10.3390/xxxxx

Table S3. Alignment Statistics of Clean Reads to the Barley Reference Genome.

Sample	Total Clean Reads (M)	Total Mapping (%)	Uniquely Mapping (%)
LEAF_0H_1	45.18	75.13	71.85
LEAF_0H_2	44.89	91.48	86.94
LEAF_12_1	43.96	92.48	85.78
LEAF_12_2	44.42	82.58	77.52
LEAF_24_1	44.39	88.27	82.55
LEAF_24_2	44.48	92.28	87.48
ROOT_0H_1	45.24	91.21	88.88
ROOT_0H_2	43.83	90.84	88.69
ROOT_12_1	45.35	91.02	89.05
ROOT_12_2	44.61	89.47	87.4
ROOT_24_1	44.35	88.42	86.27
ROOT_24_2	44.78	90.39	88.42

https://doi.org/10.3390/xxxxx

Table S4. Transcript Abundance Distribution Across Barley RNA Libraries Based on TPM Values.

TPM Range	LEAF_0H_1	LEAF_0H_2	LEAF_12_1	LEAF_12_2	LEAF_24_1	LEAF_24_2	ROOT_0H_1	ROOT_0H_2	ROOT_12_1	ROOT_12_2	ROOT_24_1	ROOT_24_2
**TPM≥ 10**	8758	8425	7510	7512	7817	8409	9807	10506	11000	11354	11107	10889
**TPM1–10**	9233	8668	9596	9854	9512	9609	9948	9689	9935	9532	9970	9994
**TPM ≤ 1**	20132	21030	21017	20757	20794	20105	18368	17928	17188	17237	17046	17240

https://doi.org/10.3390/xxxxx
